# Inhibition of cyclin‐dependent kinase 9 synergistically enhances venetoclax activity in mantle cell lymphoma

**DOI:** 10.1002/jha2.48

**Published:** 2020-08-04

**Authors:** Xiaoxian Zhao, Juraj Bodo, Ruoying Chen, Lisa Durkin, Andrew J. Souers, Darren C. Phillips, Eric D. Hsi

**Affiliations:** ^1^ Department of Laboratory Medicine Robert J. Tomsich Pathology and Laboratory Medicine Institute Cleveland Clinic Cleveland Ohio USA; ^2^ Oncology Discovery AbbVie Inc. Chicago Illinois USA

**Keywords:** CDK9 inhibitor, mantle cell lymphoma, MCL‐1, venetoclax

## Abstract

Mantle cell lymphoma (MCL) is an aggressive and largely incurable subtype of non‐Hodgkin's lymphoma. Venetoclax has demonstrated efficacy in MCL patients with relapsed or refractory disease, however response is variable and less durable than CLL. This may be the result of co‐expression of other anti‐apoptotic proteins such as MCL‐1, which is associated with both intrinsic and acquired resistance to venetoclax in B‐cell malignancies. One strategy for neutralizing MCL‐1 and other short‐lived survival factors is to inhibit CDK9, which plays a key role in transcription. Here, we report the response of MCL cell lines and primary patient samples to the combination of venetoclax and novel CDK9 inhibitors. Primary samples represented de novo patients and relapsed disease, including relapse after ibrutinib failure. Despite the diverse responses to each single agent, possibly due to variable expression of the BCL‐2 family members, venetoclax plus CDK9 inhibitors synergistically induced apoptosis in MCL cells. The synergistic effect was also confirmed via venetoclax plus a direct MCL‐1 inhibitor. Murine xenograft studies demonstrated potent in vivo efficacy of venetoclax plus CDK9 inhibitor that was superior to each agent alone. Together, this study supports clinical investigation of this combination in MCL, including in patients who have progressed on ibrutinib.

## 1 INTRODUCTION

Mantle cell lymphoma (MCL) is an incurable subtype of B‐cell NHL with a hallmark t(11;14)(q13;q32) chromosomal translocation [1]. This translocation causes deregulation of the cell cycle [2] and expansion of tumor B‐cells in the mantle zone of lymphoid follicles. The acquisition of secondary genetic alterations accounts for the aggressive behavior of some MCL cases where, despite the efficacy of first‐line treatment, the disease inevitably relapses [3, 4]. Chemoimmunotherapy regimens and allogeneic stem‐cell transplantation are commonly used to treat relapsed/refractory MCL, although there is no standard therapy [5]. Ibrutinib is currently the most effective single agent for patients ineligible for transplantation [5]. However, both inherent and acquired resistance to ibrutinib has been described [6, 7, 8, 9]. More effective treatments are therefore needed for relapsed MCL patients.

Defects in apoptosis are a hallmark of cancer cells and important for tumorigenesis and chemoresistance [10]. The involvement of pro‐survival BCL‐2 family proteins in apoptosis‐resistant tumors makes them promising targets for therapy [11]. BCL‐2 is expressed in 40‐80% of diffuse large B‐cell lymphoma (DLBCL) [12, 13, 14, 15] and MCL‐1 is present in 50% of ABC type and 30% of GCB type of DLBCL [16]. Concurrent overexpression of BCL‐2 and MCL‐1 has been described in clinical DLBCL samples [17] and other subtypes of NHL [18]. The co‐expression of anti‐apoptotic proteins in NHL samples suggests that therapeutic targeting of one of these proteins exclusively may lack efficacy in the clinic. Venetoclax shows high objective response rates in CLL as a monotherapy [19, 20, 21]. However, the single‐agent efficacy of venetoclax has not been as extensive and durable in patients with relapsed or refractory NHL [22], a collection of B‐cell malignancies where MCL‐1 is established as a factor driving resistance to venetoclax [17, 23, 24, 25]. Given the co‐expression of both BCL‐2 and MCL‐1 in subsets of NHL such as MCL [18], we hypothesized that simultaneous targeting of these proteins would afford increased efficacy in this disease.

CDK9 is a serine/threonine kinase that forms the catalytic core of the positive transcription elongation factor b. CDK9 phosphorylates Ser‐2 in the C‐terminal domain of RNA Polymerase‐II (Pol‐II), which is required for transcript elongation [26, 27, 28]. This can be blocked through small molecule CDK9 inhibition, where binding to the CDK9 subunit prevents it from interacting with the C‐terminal domain of RNA Pol‐II. CDK9 inhibition has the most immediate effect on proteins with rapid turnover rates such as XIAP and MCL‐1 [29]. Consequently, CDK9 inhibition in cell lines that depend upon functional MCL‐1 for survival results in the rapid induction of apoptosis [30, 31].

In this study, the combined effects of venetoclax with the selective CDK9 inhibitors A‐1467729 and A‐1592668 [31] were investigated in MCL cell lines and primary samples representing de novo and relapsed cases. We observed potent combination activity in both in vitro and in vivo models. Further studies deciphering the mechanism of the combined effects supported the hypothesis that MCL‐1 downregulation via CDK9 inhibition can sensitize MCL cells to BCL‐2 inhibition.

## 2 MATERIALS AND METHODS

### 2.1 Materials and treatment

Jeko‐1 cells were from DSMZ. Mino and JVM‐2 cells were from ATCC. CCMCL1 cells were obtained as described previously [32]. Lymphoma biopsies were obtained following protocols approved by the Institutional Review Board of the Cleveland Clinic. For patient samples of peripheral blood or bone marrow aspirates, mononuclear cells were isolated via Ficoll density gradient centrifugation, and PBMC or BMMC were used for further studies. For the preparation of cell suspension with lymphoma tissue specimen, briefly, razor blade was used to finely dice the tissue at first, followed by incubation with collagenase II and trypsin inhibitor. Cell suspension was then filtrated through 40 μM of cell strainer for further study. Immunoblotting antibodies are summarized in Table S1. Venetoclax [33], CDK9 inhibitors A‐1467729 and A‐1592668 [31], dinaciclib, and MCL‐1 inhibitor A‐1210477 [34] were obtained from AbbVie Inc. (North Chicago, IL). These inhibitors were dissolved in anhydrous DMSO at 10 mM and kept at −20°C for in vitro studies. Primary cells or cell lines were cultured in RPMI1640‐10% FBS medium with or without the indicated inhibitor(s) for 5 h before further experiment.

### 2.2 Apoptosis and immunoblotting

Cells were collected after treatment following BD Biosciences’ Annexin V‐PE/7‐aminoactinomycin D (7‐AAD) staining protocol (BD Biosciences, San Jose, CA). Apoptotic cells were determined by flow cytometry with a MACSQuant Analyzer 10 (Miltenyi Biotec. Auburn CA). Malignant B cells were gated with CD45, CD19, and IgG light chain. The percentage of apoptotic cells was normalized with the untreated sample. Cellular lysate preparation and immunoblotting were described previously [35].

### 2.3 Immunohistochemistry

Briefly, 4 μm of formalin‐fixed paraffin‐embedded tissue sections were used for IHC staining. Resources of antibodies, equipment, and detection systems are summarized in Table S2.

### 2.4 Xenograft study

Jeko‐1 cells (5 × 10^6^) were injected subcutaneously into NOD *scid* gamma (NOD.Cg‐Prkdc^scid^ Il2rg^tm1Wjl^/SzJ, NSG) mice on an IACUC‐approved protocol. When tumors reached approximately 3‐5 mm in length, mice were randomly assigned to each group and started treatment. Venetoclax was administered to mice by oral gavage once daily. A‐1592668 or dinaciclib, were applied to mice twice a week via oral gavage and intraperitoneal injection respectively. Formulation of A‐1592668 and venetoclax were described previously [31, 33]. Dinaciclib was dissolved in DMSO (final volume 5%), then mixed with 5% hydroxypropyl‐beta‐cyclodextrin in physiological saline (95% volume). The diameters of subcutaneous tumors were measured and tumor volume (mm^3^) was calculated as described previously [32] at the endpoints of the study or when tumors reached the maximum size allowed by IACUC.

### 2.5 Statistical analysis

The interaction between drugs was examined according to the Chou and Talalay method [36, 37] using Calcusyn (Biosoft, Cambridge, United Kingdom). Combination index (CI) values served to determine the effect of combination as synergistic (< 1), additive (= 1), or antagonistic (> 1). Spearman rank correlation analysis was performed using Statistica (StatSoft, Inc., Tulsa, OK), and significance was determined by two‐tailed Student's test.

## 3 RESULTS

### 3.1 Responses of MCL cell lines to venetoclax and CDK9 inhibitors

**FIGURE 1:**
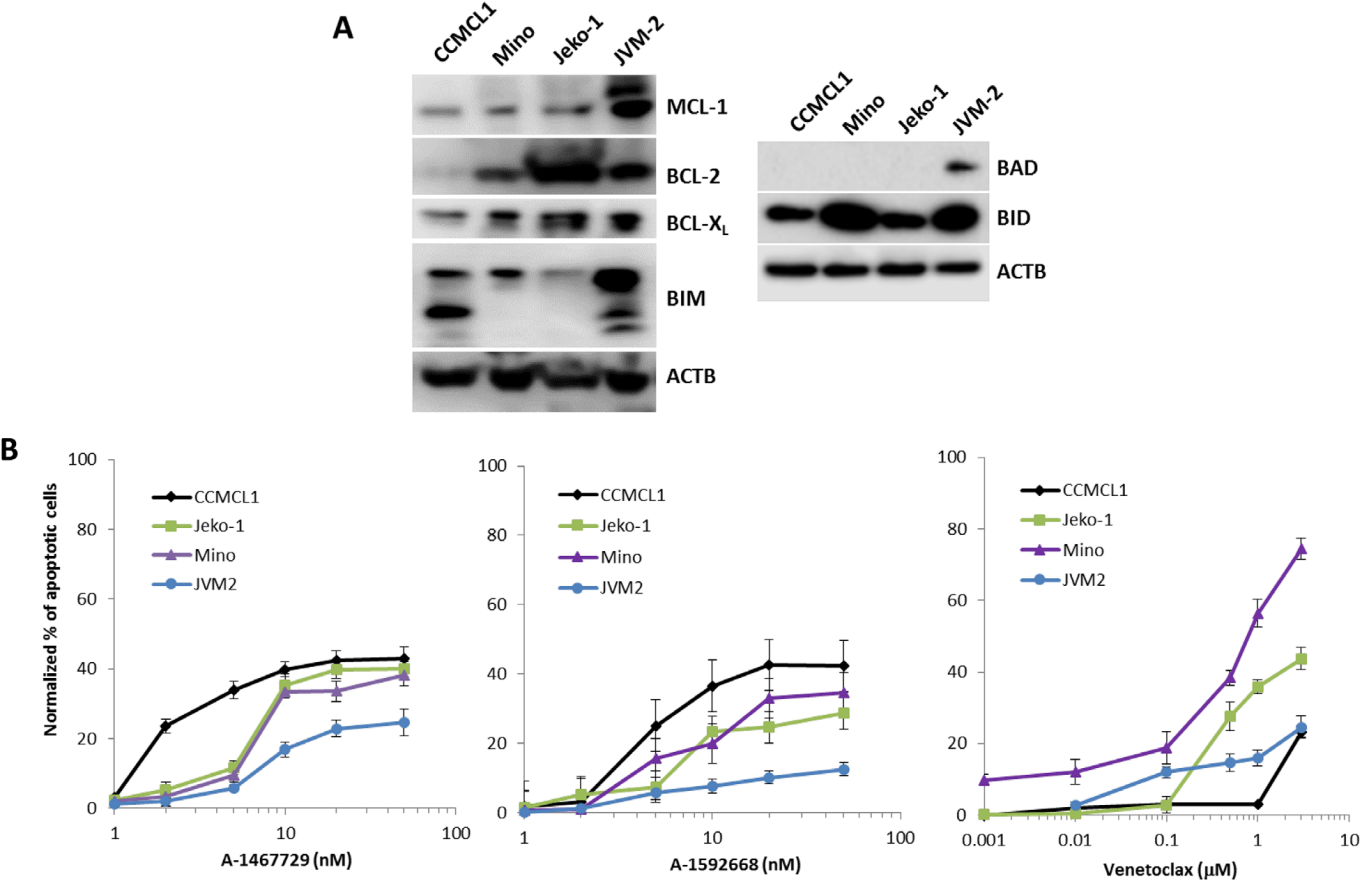
Expression of BCL‐2 family proteins in MCL cell lines and their response to venetoclax or CDK9 inhibitors. A, Immunoblotting assay of BCL‐2 family proteins utilizing β‐actin (ACTB) as a loading control. B, MCL cell lines were treated with indicated doses of A‐1467729, A‐1592668, or venetoclax for 5 h and apoptotic cells were determined by flow cytometry (Annexin‐V/7‐AAD positive population). Data are presented as the mean ± SEM of three independent experiments

NHL is characterized by heterogeneous expression of the BCL‐2 family of apoptosis regulators [17, 18], which may in part reflect the limited activity of venetoclax in DLBCL patients [22]. To understand whether this expression profile is also reflective of MCL, we first analyzed the expression of BCL‐2 family proteins in four MCL cell lines. There was a concurrent expression of BCL‐2 and BCL‐X_L_ in Mino, Jeko‐1, and JVM‐2 cells and elevated expression of MCL‐1 in JVM‐2 cells. CCMCL1 cells had almost no detectable BCL‐2. Whereas the BH3‐only protein BID was expressed in all lines, the related BH3‐only protein BAD was detectable only in JVM‐2. BIM was detectable in CCMCL1 and JVM2 cells, however only the BIM_EL_ isoform was detectable in Mino and Jeko‐1 cells (Figure 1A). Subsequent treatment of these MCL cell lines with venetoclax induced an apoptotic response profile that was reflective of the BCL‐2 family heterogeneity. CCMCL1 and JVM‐2 cells were resistant to venetoclax alone (IC_50_ > 3 μM), whereas Mino cells were more sensitive to venetoclax‐induced apoptosis and Jeko‐1 cells were less sensitive (Figure 1B). The recently described CDK9 selective inhibitors A‐1467729 and A‐1592668 [31] induced apoptosis at concentrations below 10 nM in all cell lines, with JMV‐2 cells being the least sensitive to treatment.

### 3.2 CDK9 inhibitor synergistically enhances venetoclax activity and causes rapid loss of MCL‐1

**FIGURE 2:**
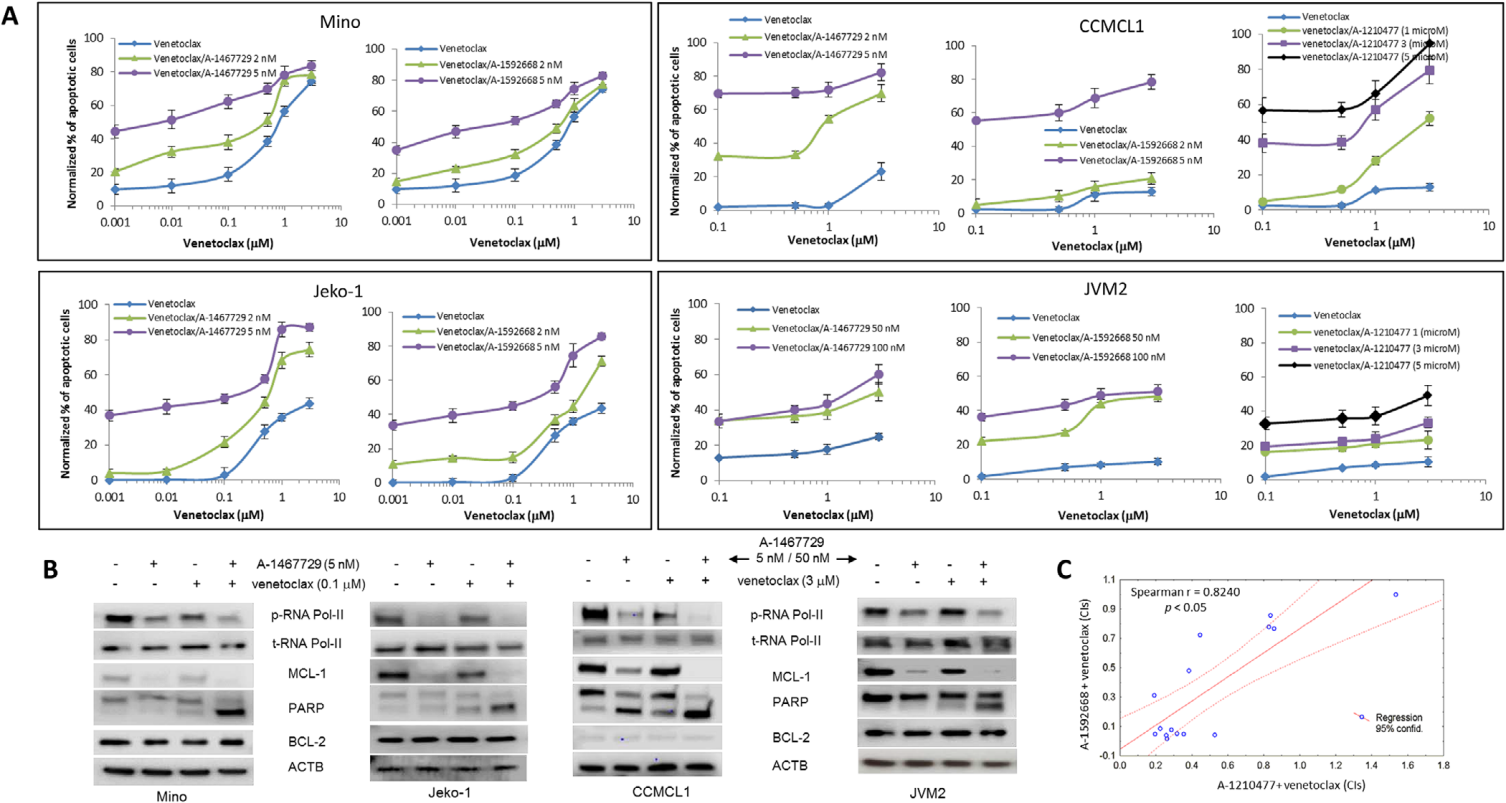
Combined effect of CDK9 inhibitor plus venetoclax or MCL‐1 inhibitor plus venetoclax in MCL cell lines. **A,** Venetoclax plus A‐1467729, A‐1592668, or A‐1210477 synergistically induces apoptosis in MCL cells. The four MCL cell lines were co‐treated with indicated inhibitors at the indicated concentrations for 5 h and apoptotic cells were determined by flow cytometry. Data are presented as the mean of three independent experiments with error bars indicating the SEM. Combination index (CI) plots are summarized in Figure S1. **B,** CDK9 inhibition reduces p‐RNA pol‐II (Ser2) and MCL‐1 expression to enhance venetoclax‐caused cleavage of PARP. Each cell lines were treated with venetoclax, A‐1467729, or venetoclax plus A‐1467729 at the indicated concentrations and times for 5 h and the impact on phospho‐RNA polymerase II (Ser2), total RNA polymerase II, PARP, BCL‐2, and MCL‐1 determined by western blot utilizing β‐actin (ACTB) as a loading control. **C,** Significant correlation (*P* < .05) between CIs of venetoclax plus A‐1592668 and venetoclax plus A‐1210477 in tested JVM‐2 and CCMCL1 cell lines

Despite the different responses to each single agent, venetoclax plus either A‐1467729 or A‐1592668 resulted in synergistic apoptosis in all MCL cell lines tested (Figure 2A; Figure S1A,B) including JVM‐2 cells, which were less sensitive to each single‐agent treatment. In concordance with the single‐agent data in which JVM‐2 cells were the least sensitive cells, the overall apoptotic effect of the combination was also lower in this cell line. The combination of venetoclax with A‐1467729 strongly enhanced PARP cleavage in all cell lines and was associated with the mechanism‐based downregulation of p‐RNApol‐II (Ser2) and MCL‐1 whereas BCL‐2 levels were unaffected (Figure 2B).

### 3.3 Direct MCL‐1 inhibition has similar effects as CDK9 inhibition

To confirm that CDK9 inhibitor‐induced reduction of MCL‐1 protein contributed to the synergy between venetoclax and A‐1467729/A‐1592668, the MCL‐1 inhibitor A‐1210477 was tested in combination with venetoclax. As shown in Figure 2A and Figure S1B,C, venetoclax plus A‐1210477 demonstrated similar synergistic effects on apoptosis induction as the combination of venetoclax plus CDK9 inhibitors in CCMCL1 and JVM2 cells. Figure 2C represents a linear analysis between CIs of venetoclax plus MCL‐1 inhibitor and venetoclax plus CDK9 inhibitor. Spearman's rank correlation analysis showed a significant correlation (*P* < .05) between the CI values in JVM2 and CCMCL1 cell lines.

### 3.4 CDK9 inhibitor or MCL‐1 inhibitor enhances venetoclax activity in primary cells

**FIGURE 3:**
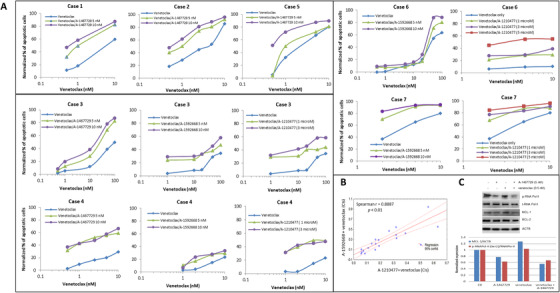
Interactions of venetoclax plus CDK9 inhibitors or venetoclax plus MCL‐1 inhibitor in primary MCL cells. A, Primary MCL cells were co‐treated for 5 h with venetoclax plus A‐1467729; venetoclax plus A‐1592668 or venetoclax plus A‐1210477 at the indicated concentrations and the effect on apoptosis was determined by flow cytometry. B, Significant correlation of CIs between venetoclax plus A‐1592668 and venetoclax plus A‐1210477 were subsequently determined (*P* < .01). C, A‐1467729 reduces p‐RNA pol‐II (Ser‐2) and MCL‐1 expression in primary MCL cells (case 2). Immunoblotting of phospho‐RNA pol‐II (Ser2), total RNA pol‐II, MCL‐1, BCL‐2, and β‐actin (ACTB) were performed after treatment without or with A‐1467729 (5 nM), venetoclax (0.5 nM), or their combination for 5 h. The densitometries of phospho‐RNA pol‐II (Ser2) bands or MCL‐1 bands were normalized with total RNA Pol‐II or with ACTB, respectively

Seven primary MCL cases (Table 1) were tested for their *ex vivo* sensitivity to venetoclax with or without CDK9 inhibition. Venetoclax alone at doses of < 10 nM caused apoptosis in cases 1, 2, 4, 5, and 7, while five‐ to tenfold higher doses of venetoclax were required for significant apoptosis induction in tumor cells from case 3 and 6 (Figure 3A). Similar to the cell lines, CDK9 inhibition synergistically enhanced venetoclax‐induced apoptosis in all primary samples (Figure 3A), with CI values < 1 for all combinations except those of case 6 (a blastoid MCL) in which only a subset of combination doses displayed synergy (Figure S2). Cases 3 and 4 were also compared for their responses to venetoclax plus A‐1592668 and venetoclax plus A‐1210477. As shown in Figure 3A, both combinations displayed synergistic effects and there was a significant correlation between CIs of venetoclax plus A‐1592668 and venetoclax plus A‐1210477 (Figure 3B, *P* < .01). Similar data were collected for case 7 (Figure 3A). Case 6 was the sole example in which the synergistic effects were detectable only at higher doses of venetoclax. Interestingly, a robust synergistic effect was observed for this case upon venetoclax plus A‐1210477 (Figure 3A). Quantification of immunoblotting of case 2 showed that CDK9 inhibition caused a loss of 38% of phospho‐RNA pol‐II (Ser‐2) in comparison with total RNA pol‐II, and a loss of 23% of MCL‐1 protein in comparison with β‐actin in the primary MCL cells (Figure 3C).

**TABLE 1:** Primary MCL cases used in this study

Case #	Sample	Age/gender	WBC (x10^9^/L)	% of lymphoma cells	Diagnosis	Cytogenetics
1	Bone marrow aspirate	71/F	34.9	77	New diagnosed MCL	46,XX,+8,add(9)(p13)‐11,der(14)t(11;14)(q13;q32),add(12)(q24.1),add(19)(p10)[3]/46,XX[17]
2	Peripheral blood	71/M	231	84	Relapsed MCL after therapies with rituxan, bortezomib, bendamustine and ibrutinib	42‐44,XY,der(3)t(3;12)(q27;q13),t(11;14)(q13;q32),‐13,‐14,add(15)(q26),‐18,add(19)(q13.3),‐20,‐22,‐22,+2‐6mar[cp6]/46,XY[14]
3	Peripheral blood	58/M	851	99	Relapsed MCL with failure of bendamustine, rituximab, hydroxyurea, bortezomib, cyclophosphamide, doxorubicin, vincristine and prednisone	47,XY,t(Y;12)(p35;q12),add(1)(p32),del(2)(q11.2q31),der(2)t(2;11)(q13q13),add(5)(q11.2q31),t(8;22)(q24;q11.2),del(9)(q13q22),add(11)(p15),der(11)t(11;14)(q13q32),add(12)(p11.1),del(20)(q11q13)[20]
4	Spleen biopsy	63/M	N/A	90	Blastoid MCL	45,XY,add(4)(q35),add(8)(p23),‐9,‐19,+mar[10]/44,XY,add(8)(p23),‐9,‐10,‐19,add(22)(q13),+mar[9]/46,XY[1]
5	Peripheral blood	76/M	7.4	46	Blastoid MCL	N/A (FISH: IGH/CCND1 +)
6	Bone marrow aspirate	64/M	29.5	62	Persistent/recurrent MCL, blastoid variant	46,XY[9]
7	Peripheral blood	69/M	78.3	83	Persistent/recurrent MCL	46,XY,del(6)(q21q25),add(9)(p13),‐10,t(11;14)(q13;q32),+mar[3]/46,XY,t(3;11)(q21;q 23)[3]/46,XY[16]

### 3.5 Immunohistochemistry

**FIGURE 4:**
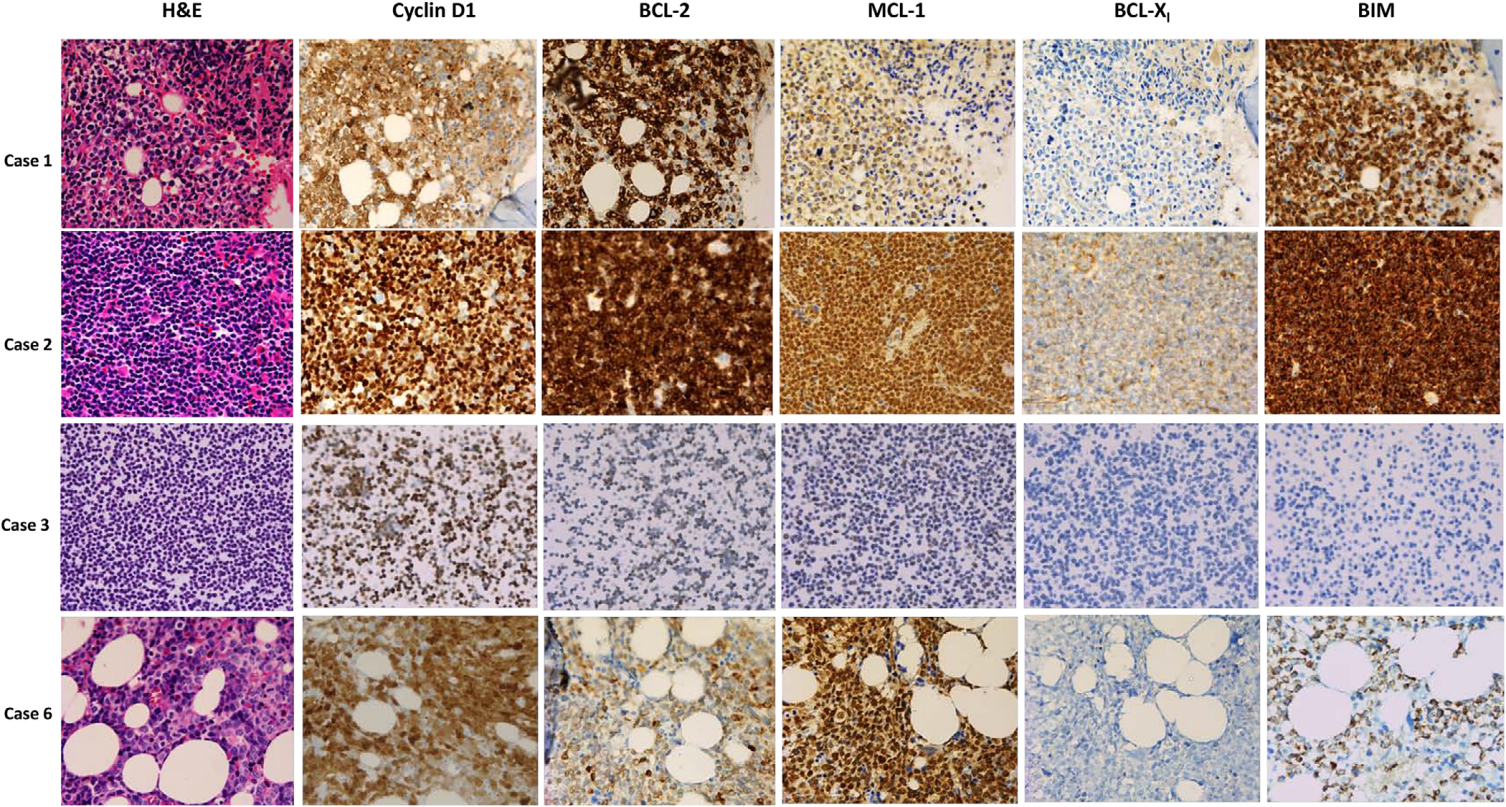
Immunohistochemical staining. Tissue sections of MCL cases 1, 2, 3, and 6 were used for IHC with indicated antibodies. Cases 1 and 6: formalin fixed paraffin embedded bone marrow tissues; case 2: formalin fixed paraffin embedded lymph node tissue; case 3: cytospin slide of isolated PBMC. Detailed information on primary antibodies or detection methods are presented in Table S2

To examine the correlation between BCL‐2 and MCL‐1 expression and sensitivity of primary samples to the treatment, we performed immunohistochemistry staining on cases with available tissues (1, 2, and 6) or cytospin (case 3) blocks. As shown in Figure 4, all cases were positive for cyclin D1. Concurrent expression of BCL‐2 and MCL‐1 were detected in all cases but with diverse expression pattern among the cases. Compared to BCL‐2 or MCL‐1 expression levels, BCL‐X_L_ protein was either undetectable (cases 3 and 6) or relatively weak expression. BIM was strongly positive in cases 1 and 2, weakly positive in case 6, and undetectable in case 3. This lack of BIM expression in case 3 was consistent with our previous SNP microarray data [32], which demonstrated BIM deletion on chromosome 2q13. As summarized in Table 1, case 3 was a relapsed MCL with multiple drug failures and a complex karyotype, including MYC rearrangement. Together, this may explain the lack of response to venetoclax. Compared to cases 1 and 2, case 6 displayed weak BCL‐2 but stronger MCL‐1 expression, which is consistent with the weak response of these cells to venetoclax yet robust sensitivity to the combination of venetoclax plus MCL‐1 inhibitor A‐1210477 (Figure 3A).

### 3.6 Antitumor efficacy of venetoclax plus CDK9 inhibition in a xenograft model of MCL

**FIGURE 5:**
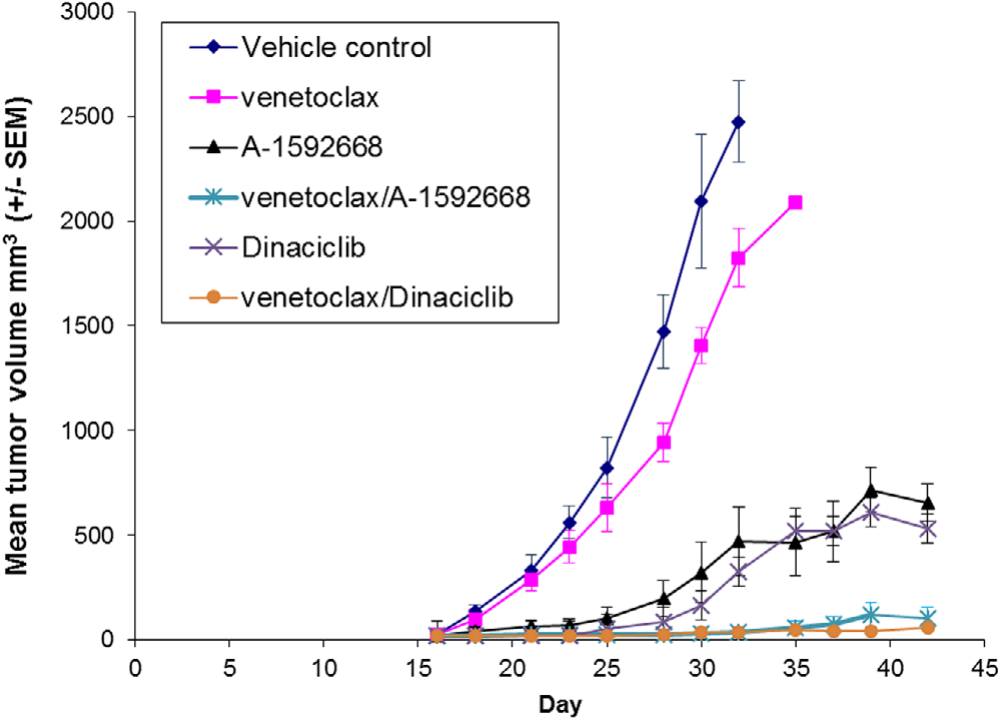
The combination of venetoclax with CDK9 inhibitors provides added benefit over either agent alone in a xenograft model of MCL. NSG mice bearing engrafted Jeko‐1 cells were treated with A‐1592668 (4 mg/kg, twice a week with oral gavage), dinaciclib (30 mg/kg, twice a week with intraperitoneal injection), venetoclax (50 mg/kg, everyday with oral gavage), or venetoclax in combination with A‐1592668 or dinaciclib, and the diameters of subcutaneous tumors were measured. Data are presented as the mean tumor volume ± SEM obtained from six mice per treatment group. Venetoclax treated mice had less tumor burden than vehicle arm on day 28 or later (*P* < .05). A‐1592668, dinaciclib, or their combinations with venetoclax significantly reduced the tumor burden compared with control mice with *P*‐values of .024, .001, < .001, and < .001, respectively. Venetoclax plus A‐1592668 or dinaciclib further minimize the tumor volumes than A‐1592668 or dinaciclib alone with *P*‐values of .0014 and .0048, respectively. There was no significant difference between A‐1592668 and dinaciclib (*P* = .50) or between venetoclax plus A‐1592668 versus venetoclax plus dinaciclib (*P* = .23).

To examine the effects of concomitant inhibition of BCL‐2 and CDK9 in vivo, we evaluated the combination of venetoclax plus A‐1592668 or dinaciclib, a pan CDK inhibitor that has been tested in clinical trials for advanced malignancies [38, 39]. NSG mice were inoculated with Jeko‐1 cells, then randomly divided into six groups with six mice per group when tumors reached 25 mm^3^. Venetoclax was dosed at 50 mg/kg/day, while A‐1592668 and dinaciclib were dosed at 4 mg/kg or 30 mg/kg twice a week, respectively. All agents were well tolerated as monotherapy or in combination. As shown in Figure 5, treatment with each single agent reduced the tumor burden compared to the control. Venetoclax plus A‐1592668 or venetoclax plus dinaciclib co‐treatment robustly inhibited the growth of Jeko‐1 tumors during 4 weeks of treatment and provided a significant survival advantage over either agent alone or the vehicle control arm (*P* < .05). These data demonstrate that the combination of venetoclax plus CDK9 inhibition is effective against MCL in vivo.

## 4 DISCUSSION

Venetoclax is a potent and selective BCL‐2 inhibitor that has demonstrated high objective response rates as a monotherapy in patients with CLL, a B‐cell malignancy that possesses a strong dependence on BCL‐2 for survival. While venetoclax has shown objective responses in a number of NHL subtypes as monotherapy, the overall response rates and durability were not as high as in CLL [19, 22]. Co‐expression and co‐dependence on multiple BCL‐2 family proteins in NHLs may act as a resistance factor to venetoclax. Agents targeting MCL‐1 through transcriptional repression have shown robust combination activity with venetoclax in disease models that are co‐dependent on both proteins [17, 25]. The combination of venetoclax with CDK9 inhibitors, therefore, remains a promising strategy for improving outcomes in NHL patients, including those with MCL.

Herein, we evaluated the expression of BCL‐2 family members in a panel of MCL cell lines and primary patient samples, and related their expression patterns to venetoclax activity in vitro. As with other subtypes of NHL [17, 18, 25], the expression of the anti‐apoptotic BCL‐2 family members varied widely in MCL cells. Relatively low BCL‐2 expression was observed in the CCMCL1 cell line and in primary MCL cases 3 and 6, which was associated with less sensitivity to venetoclax monotherapy. MCL‐1 is a well described resistance factor to venetoclax. Notably, high MCL‐1 expression was observed in the MCL cell line JVM‐2 and primary patient sample case 6, both of which lacked sensitivity to venetoclax treatment.

CDK9 inhibition represents a therapeutically tractable approach for reducing the expression of short‐lived survival factors such as MCL‐1 and subsequently driving apoptosis in tumor cells that depend upon these factors for survival [17, 25, 31]. Although the CDK9 selective inhibitors A‐1467729 and A‐1592668 had mild single‐agent activity in MCL cell lines and primary patient samples, broad synergy with venetoclax was observed across all MCL cell systems studied that was related to the loss in RNApoll‐II phosphorylation and the concomitant reduction in MCL‐1 protein levels. In vivo xenograft studies confirmed the robust combined activities of venetoclax plus CDK9 inhibition against MCL cells. While the effects of CDK9 inhibition can be manifold and not solely due to decreased MCL‐1 levels [29, 40], the breadth of expression and demonstration that direct MCL‐1 inhibition with A‐1210477 phenocopies the CDK9 inhibition implicates the former as a relevant target in MCL. This is consistent with a recent study demonstrating that co‐targeting BCL‐2 and MCL‐1 with selective small molecule inhibitors is efficacious in patient‐derived xenograft models of relapsed MCL [41].

Expression of the pro‐survival protein BCL‐X_L_, and downregulation or loss of the pro‐death protein BIM are additional mechanisms that can limit the activity of venetoclax [23, 41, 42]. Although MCL cell lines expressed BCL‐X_L_, the primary MCL patient samples had low or undetectable levels of BCL‐X_L_ as determined by IHC. MCL cases 3 and 6 were negative or low for BIM expression, which may have contributed to the limited single‐agent venetoclax activity observed along with low expression of BCL‐2. Despite these observations, concomitant inhibition of BCL‐2 and CDK9 was effective in inducing apoptosis in these samples. Considering the overall response of NHL patients to venetoclax monotherapy [22], these data collectively indicate that combination with CDK9 inhibitors may overcome mechanism of clinically relevant resistance to venetoclax monotherapy.

Genomic characterization of MCL samples identified the loss of chromosome 9p21.1‐p24.3 containing *SMARCA2, ARID2*, and *SMARCA4* genes in MCL patients who failed to respond to combined ibrutinib and venetoclax, suggesting that mutations in the SWI‐SNF chromatin‐remodeling complex mediate resistance to this combination in MCL [43]. The impact of *SWI‐SNF* mutations on the response to other targeted therapeutics have not been investigated. Among the seven primary MCL cases we studied, none had received prior venetoclax therapy and therefore the sensitivity or resistance of these patients to this agent clinically is unknown. There are cytogenetic data for six cases (Table 1). While no deletions of chromosome 9p21.1‐p24.3 were detected in five cases, case #4 showed a copy loss of chromosome 9 in 19 of 20 cells analyzed. Since the combination of venetoclax plus CDK9 inhibition induced greater apoptosis relative to either as monotherapy in the seven MCL cases, which have distinct cytogenetic backgrounds, it would be interesting to test their efficacy in MCL samples harboring *SWI‐SNF* mutations. Indeed an unbiased broad‐scale genomic/transcriptomic approach to better understand mechanisms of action and predictive markers for response in a large number of cases would be a logical next step.

In summary, despite heterogeneous response to venetoclax or CDK9 inhibitors as single agents, MCL cell lines and primary samples from untreated and relapsed aggressive cases were sensitive to the concomitant inhibition of BCL‐2 and CDK9. The efficacy of the venetoclax plus MCL‐1 inhibitor combination significantly correlated with that of the venetoclax plus CDK9 inhibition, supporting the hypothesis that enhanced antitumor activity can be achieved when venetoclax is combined with agents that inhibit MCL‐1 or repress its expression. Furthermore, the *in vitro* efficacy derived from this combination was recapitulated in a mouse xenograft model. These findings support the clinical evaluation of venetoclax in combination with CDK9 or MCL‐1 inhibitors in MCL, including ibrutinib‐resistant MCL.

## AUTHOR CONTRIBUTIONS

XZ and EDH are the principal investigators and take primary responsibility for the manuscript. XZ, RC, and LD performed laboratory work for this study. JB performed the analysis of data. XZ and EDH coordinated the research. XZ, JB, AS, DP, and EDH contributed to the experimental design and writing of the manuscript.

## CONFLICT OF INTEREST

AS and DP are employees of AbbVie Inc. and are stockholders. AbbVie Inc. participated in the interpretation of data, review, and approval of this publication. Other authors have no conflict of interest to disclose.

## Supporting information


**Figure 1** Combination index (CI) plots of each cell line. The synergy effects of each indicated combinations in each cell lines were calculated by using Calcusyn (Biosoft, Cambridge, United Kingdom). Y axis represents CI values and X axis represents effect levels.
**Figure 2** CI plots of tested combination of indicated agents in primary MCL samples

**Table S1** Immunoblotting antibodies
**Table S2** IHC antibodies and detection methods
