# Supplementary material for: Inhibition of cyclin‐dependent kinase 9 synergistically enhances venetoclax activity in mantle cell lymphoma
Source: EJHaem. 2020 Aug 4;1(1):161–9. doi: 10.1002/jha2.48 (PMC9176003; doi:10.1002/jha2.48)
Supplement: Supplementary file 1 — Figure 1 Combination index (CI) plots of each cell line. The synergy effects of each indicated combinations in each cell lines were calculated by using Calcusyn (Biosoft, Cambridge, United Kingdom). Y axis represents CI values and X axis represents effect levels. Figure 2 CI plots of tested combination of indicated agents in primary MCL samples [file JHA2-1-161-s001.pptx]

## Slide 1
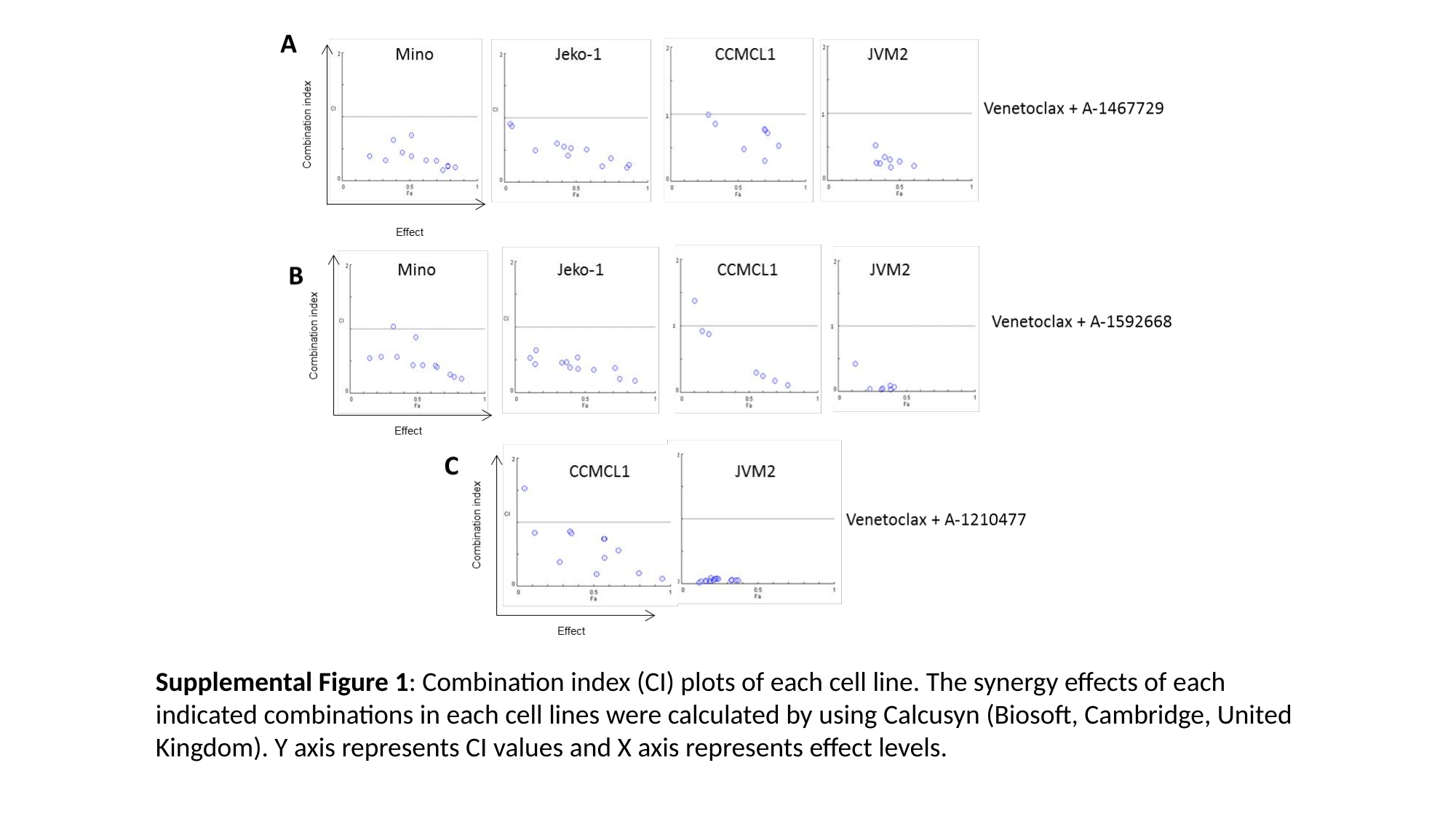

Supplemental Figure 1: Combination index (CI) plots of each cell line. The synergy effects of each
indicated combinations in each cell lines were calculated by using Calcusyn (Biosoft, Cambridge, United
Kingdom). Y axis represents CI values and X axis represents effect levels.

## Slide 2
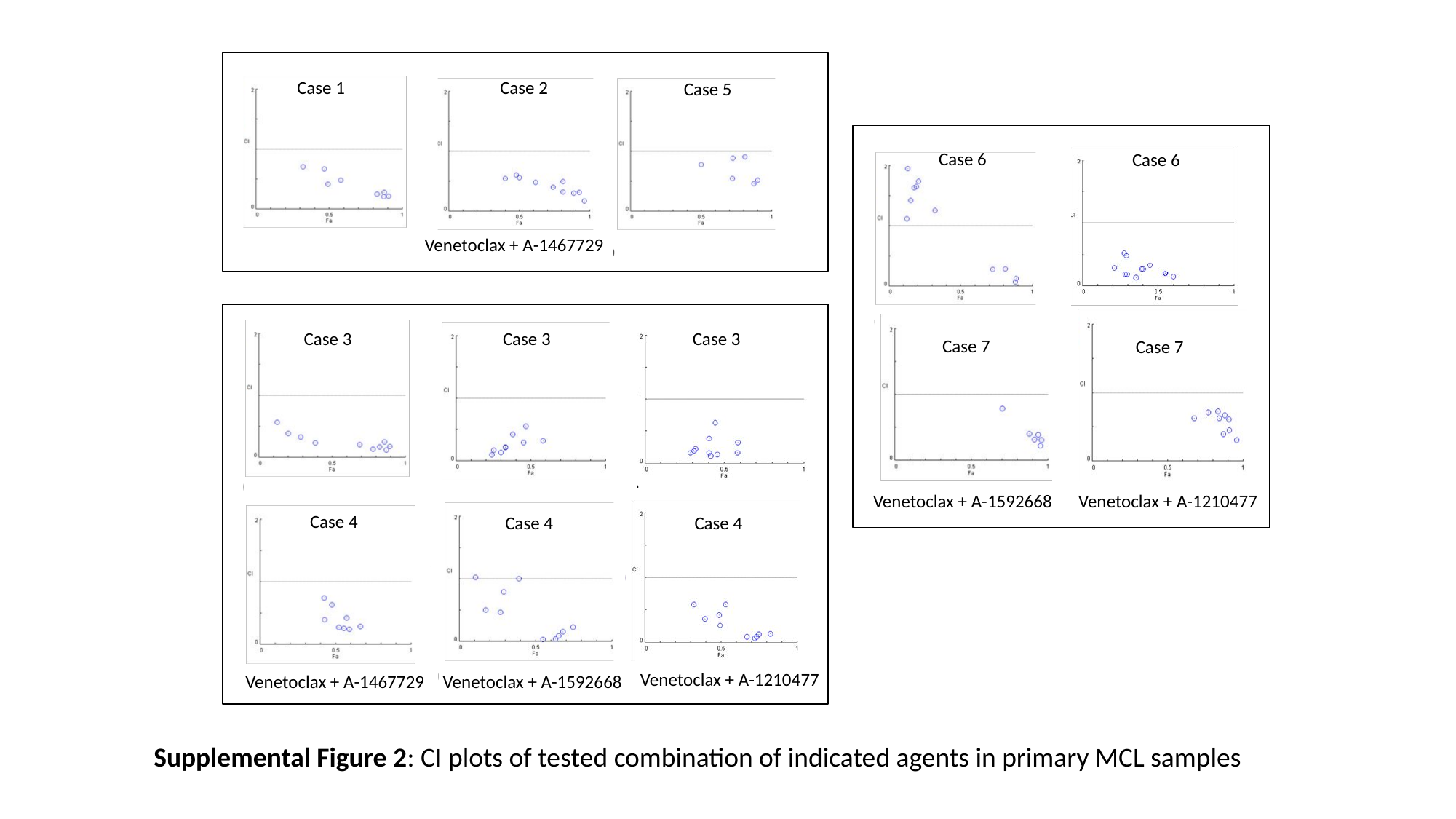

Case 1
Case 2
Case 5
Venetoclax + A-1467729
Case 6
Case 6
Case 7
Case 7
Venetoclax + A-1210477
Venetoclax + A-1592668
Case 3
Case 3
Case 3
Case 4
Case 4
Case 4
Venetoclax + A-1210477
Venetoclax + A-1592668
Venetoclax + A-1467729
Supplemental Figure 2: CI plots of tested combination of indicated agents in primary MCL samples
