# Supplementary material for: Inhibition of cyclin‐dependent kinase 9 synergistically enhances venetoclax activity in mantle cell lymphoma
Source: EJHaem. 2020 Aug 4;1(1):161–9. doi: 10.1002/jha2.48 (PMC9176003; doi:10.1002/jha2.48)
Supplement: Supplementary file 2 — Table S1 Immunoblotting antibodies Table S2 IHC antibodies and detection methods [file JHA2-1-161-s002.docx]

**Supplemental Table 1: Immunoblotting antibodies**

**Supplemental Table 2: IHC antibodies and detection methods**
